# Designing nanomaterials with desired mechanical properties by constraining the evolution of their grain shapes

**DOI:** 10.1186/1556-276X-6-585

**Published:** 2011-11-08

**Authors:** Thomas Bobga Tengen

**Affiliations:** 1 Department of Industrial Engineering and Operations Management, Faculty of Engineering and Technology, Vaal University of Technology, Private Bag X021, Vanderbijlpark 1900, South Africa

**Keywords:** grain shape, grain strain, strain rate, grain size, grain form functions, grain growth, nanomaterials' mechanical properties

## Abstract

Grain shapes are acknowledged to impact nanomaterials' overall properties. Research works on this issue include grain-elongation and grain-strain measurements and their impacts on nanomaterials' mechanical properties. This paper proposes a stochastic model for grain strain undergoing severe plastic deformation. Most models deal with equivalent radii assuming that nanomaterials' grains are spherical. These models neglect true grain shapes. This paper also proposes a theoretical approach of extending existing models by considering grain shape distribution during stochastic design and modelling of nanomaterials' constituent structures and mechanical properties. This is achieved by introducing grain 'form'. Example 'forms' for 2-D and 3-D grains are proposed. From the definitions of form, strain and Hall-Petch-Relationship to Reversed-Hall-Petch-Relationship, data obtained for nanomaterials' grain size and conventional materials' properties are sufficient for analysis. Proposed extended models are solved simultaneously and tested with grain growth data. It is shown that the nature of form evolution depends on form choice and dimensional space. Long-run results reveal that grain boundary migration process causes grains to become spherical, grain rotation coalescence makes them deviate away from becoming spherical and they initially deviate away from becoming spherical before converging into spherical ones due to the TOTAL process. Percentage deviations from spherical grains depend on dimensional space and form: 0% minimum and 100% maximum deviations were observed. It is shown that the plots for grain shape functions lie above the spherical (control) value of 1 in 2-D grains for all considered grain growth mechanisms. Some plots lie above the spherical value, and others approach the spherical value before deviating below it when dealing with 3-D grains. The physical interpretations of these variations are explained from elementary principles about the different grain growth mechanisms. It is observed that materials whose grains deviate further away from the spherical ones have more enhanced properties, while materials with spherical grains have lesser properties. It is observed that there exist critical states beyond which Hall-Petch Relationship changes to Reversed Hall-Petch Relationship. It can be concluded that if grain shapes in nanomaterials are constrained in the way they evolve, then nanomaterials with desired properties can be designed.

## Introduction

Nanomaterials are a new brand of materials with noble properties. Nowadays, this class of materials is attracting continuous attention. This is due to the fact that their nanostructures can be characterised. This includes 2-D and 3-D characterisation. Due to the fact that during characterisation, most observations are made on cutting planes through sections of the materials; some of the 3-D characterisations have been done [1-6] by reconstruction from 2-D images, an approach that is also attracting some attention. After characterisations, nanomaterials can be classified into various groups depending on characteristics of the nanostructures and the nanomaterials' overall properties.

A lot of attempts have been made to classify nanostructured materials. This includes classification according to their dimensionality (shape) by Siegel [7] into 0-D nanoclusters, 1-D multilayers, 2-D nanograined layers and 3-D equiaxed bulk solids. Further classification was done by Gleiter [8] based on 'partial/complete' permutations of their composition; morphology and distribution of nanocrystalline components; rod-shaped, layer-shaped and equiaxed shape grains.

From the above classification efforts, most polycrystalline nanomaterials may be viewed as being built up by successive addition of inclusions of random sizes and shapes [8]. The examples of these inclusions include grains, pores and cracks. The sizes of these inclusions belong to the nanometre range of length scale in nanomaterials (i.e. this is the definition of nanomaterials). Though the shapes of the inclusions in nanomaterials may be completely different from those in coarse-grain (or conventional) materials, materials are called nanomaterials because of their reduced grain sizes alone, and grain shapes do not play any role in this definition. It should be emphasised that the 'true' shape of an inclusion *should not *depend on the orientation or location of the cutting plane on which these inclusions are observed under microscopes.

Nanomaterials have been proven to have enhanced overall properties such as conductivity, elasticity, permeability, stress, strain and/or energy (there are numerous reports in the literature on this fact). The overall mechanical properties of nanomaterials have also been proven to be quite different due to the random size and shape of the building blocks (or inclusions) whose mean values and dispersions may not be the same [8-15]. To elucidate, firstly, nanomaterials (from the same initial sample type) produced through different processing routes to the same mean grain size may have different grain size dispersion and as such may exhibit different material properties [9-11]. Secondly, different nanomaterials having different mean grain sizes and different grain size dispersions may possess the same material properties [9,10]. The conclusion following the last two (i.e. first and second) observations is that both mean grain size and grain size dispersion should simultaneously be used when designing and modelling nanomaterials' mechanical properties (i.e. grain size distribution is important) [9,10,13]. While remarkable success has been achieved on the concurrent employment of mean grain size and grain size dispersion in modelling and designing of nanomaterials' mechanical properties, it has also been, thirdly, acknowledged that nanomaterials from similar samples produced to the same mean grain size and the same dispersion may have different mechanical properties [9-11,13]. This may be due to the neglect of other grain characterisation parameters such as grain shapes and grain boundary structure (e.g., low-angle versus high-angle grain boundaries) [13] and the existence in the material of very small pores commonly referred to as nanovoid which are below the detection limits of density measurements and can only be inferred by positron annihilation [13,16,17]. It is reported that not accounting properly for the possible direct effects of such processing-induced defects on the mechanical response has led to numerous contradictory results in the published literature [13,18,19]. Upon inferring the existence of nanovoids, its size effects on the material properties can then be dealt with. Thus, the major challenge is with the impact of the inclusions' shapes on the material properties. Hence, simultaneously employing grain shape and size distributions during the modelling and designing of nanomaterials is vital when trying to further resolve the controversial observations. This leads to the main objective of the present paper which is to study the impact of grain shape distribution on the mechanical properties of nanomaterials.

Many research works have been done in an attempt to address the controversial issue presented in the paragraph above. This includes grain-elongation and grain-strain measurements and their impacts on the mechanical properties of nanomaterials. Most models for the relationships between grain size, grain strain and grain elongation and yield stress have been obtained by curve fittings of experimental data. The models deal 'directly' with equivalent radii of grain sizes that assume that the grains in nanomaterials are spherical, thus neglecting their true shapes. In the present paper, a stochastic model of grain strain undergoing severe plastic deformation is (theoretically) proposed from the first principle. Furthermore, a proposal is made on a theoretical approach to extend the existing models to incorporate the grain shape distribution function during the stochastic design and modelling of nanomaterials' constituent structures and yield stress. This is achieved by introducing the shape (or form) function, a knowledge gained from Stoyan [20]. Form is the relationship between grain size and shape. Since form function depends on the type of investigation under consideration and for the sake of validation of proposed models, some (example) shape functions for 2-D and 3-D grains are proposed and tested. 2-D and 3-D are the dimensional spaces that are mostly dealt with in experiments and simulations. To be able to identify the issues raised in the previous paragraph, one has to study the evolution/deformation (either during refinement or growth) of grains in nanomaterials. The enormous success previously obtained [9-11,21-23] from designing and modelling different mechanisms of grain growth and mechanical properties of nanomaterials is used in the present paper. Considering the fact that grain size is related to grain strain, grain elongation and grain yield stress, the approach presented in this report leads to simultaneous or transitional relationships between grain strain, shapes/elongations and the mechanical properties. From the definitions of form, strain, strain rate and Hall-Petch Relationship (HPR) to Reversed Hall-Petch Relationship (RHPR), data obtained for grain size and overall properties are sufficient for the current analysis. The proposed extended models are solved simultaneously and tested with the data from grain growth in nanocrystalline aluminium samples [21-23]. Theoretical analyses are done so as to anticipate how the evolution of some grain shapes towards and away from spherical grains impacts the materials' mechanical properties. This is because the modelling of nanomaterials' properties using grain sizes employs an equivalent radius that assumes that the grains are spherical in shape.

## Method

The 'main' parameter in this report is the form or shape function. It is to be emphasised that the form function is not unique for any particular shape. This implies that the result may vary with different choices of the form parameter. The choice of form parameter should depend on the type of investigation under consideration (i.e. whether the parameter characterises the parking properties or local roughness or chemical properties). An example of the form function of a 2-D grain × adopted from Stoyan [20] is

(1) $$f\left(X\right)=\frac{\text{area of X}}{{\left(\text{perimeter of X}\right)}^{2}}$$

From geometric and mathematical considerations, the shape function or form function *f(X) ≠ K*, with equality when the grains are circular, where *K *depends on the type of shape under consideration (e.g. *K *= 1/4π for circular grains). Careful study of Equation 1 reveals that *f(X) *is a dimensionless quantity. Similarly, it can be deduced that for 3-D grains (i.e. real world problems), the expressions of form function that may be used are

(2.1) $$g\left(X\right)=\frac{{\left(\text{Volume of X}\right)}^{2}}{{\left(\text{Area of X}\right)}^{3}}\neq K^{\prime }$$

(2.2) $$h\left(X\right)=\frac{\text{Volume of X}}{{\left(\text{perimeter of X}\right)}^{3}}\neq K^{\prime \prime }$$

with equality when the grains are spherical (e.g. *K*' = 1/(4(3^2^)*π*) = 1/(36*π*) for spherical grains). Thus, the evolution of grains of various shapes towards circular or spherical ones implies that *f*(*X*)/(*K*) or *g*(*X*)/(*K*') or *h(X)/(K″) *should respectively approach 1. This is an important observation which serves as constraint during modelling and should be the basis to analyse the deviation from spherical shapes. For the purpose of fluent communication in this report, *g(X) *and *h(X) *found in Equation 2 are called 3-D_Area _and 3-D_Perimeter_, respectively. This is because the 3-D volumes are made dimensionless by normalisation with area and perimeter, respectively.

To be consistent with previous results obtained using grain sizes in nanomaterials [[21-23], and references therein], the radius vector should be used to describe the contour of the grains as opposed to the use of tangent-angle functions, support functions or cross-section functions. The use of radius-vector functions should lead to Fourier series with Fourier coefficients serving as parameters. Further analysis results in information about individual grains which can then be averaged in some sense, e.g. by using the theory of compound Poisson process. Grains in nanomaterials do not have independent behaviours. Their characteristics combine with those of other neighbouring grains to yield the properties of the entire nanomaterials. Thus, the 'local-properties' approach leading to the use of Fourier series and Fourier coefficients is not pursued in this report. Rather, the global statistics is dealt with.

It should be emphasised that from shape consideration, the perimeter, area, volume and shape functions of a grain are statistically independent. Thus, the statistical moments of Equations 1 and 2 split into independent moments. The evolution of the mean grain size (i.e. size may be radius, area or volume) during grain growth has been extensively studied [[21-23], and references therein]. The evolution of grain size due to curvature-driven grain boundary migration [GBM], mis-orientation angle-driven grain rotation coalescence [GRC] mechanism and a combination of both mechanisms (i.e. TOTAL process) is given by [21-23],

(3) $$dr=M\left(r,T\right)\left(\frac{1}{r_{c}}-\frac{1}{r}\right)dt+D\sqrt{r}d\text{W}\left(t\right)+\left(1+a\right)rd\text{N}t.$$

In Equation 3, *M *(*r*, *T*) is the temperature-dependent grain boundary [GB] mobility function, *r*_c _is local critical grain size, *a *and *D *are constants, *d*W*(t) *is increment of the Wiener process and *d*N*(t) *is the number of coalescence events within an infinitesimal time interval. The evolution of nanomaterials' grain yield stress undergoing plastic deformation is given as [9-12]

(4) $$d\sigma \left(r,t\right)=\left\{\left(-\frac{A}{2r^{3∕2}}+\frac{B}{r^{2}}+\frac{3C}{2r^{5∕2}}\right)M_{\text{mig}}\left(\frac{1}{r_{\text{c}}}-\frac{1}{r}\right)+D^{2}\left[\frac{3A}{8r^{3∕2}}-\frac{B}{r^{2}}-\frac{15C}{8r^{5∕2}}\right]\right\}dt+D\left\{-\frac{A}{2r}+\frac{B}{r^{3∕2}}+\frac{3C}{2r^{2}}\right\}d\text{W}\left(T\right)+\left\{\frac{E}{r^{1∕2}}-\frac{F}{r}-\frac{G}{r^{3∕2}}\right\}d\text{N}\left(t\right)$$

where *E *= *A*(*b*-1), *F *= *B*(*b*^2^-1), *G *= *C *(*b*^3^-1) and *b *= (2 + *a*)^-1/2^, *A *= *K*_d _is the Hall-Petch Relationship proportionality constant, *B *= *K*_t _[2*hH*_m_/*RT*_r_], *C *= *K*_d_[2*hH*_m_/*RT*_r_], *K*_t _is a constant, *h *is the atomic diameter, *H*_m _is the conventional material melting enthalpy, *R *is the ideal gas constant, *T*_r _is the room temperature, *K*_d _> 100*K*_t _and σ_0 _> 10*K*_t_. On the right hand side of Equation 4, the first term accounts for the change of individual grain yield stress due to GBM processes, the second term accounts for the random fluctuation in grain yield stress due to GBM mechanisms and the last term accounts for the change in grain yield stress due to GRC mechanisms.

From the first principle, strain *ε *is defined as change in size with respect to 'original' size, i.e. $\in =\frac{\Delta r}{r}=\frac{dr}{r}$. This implies that the increment of grain strain should be given by *dε *= *d*(*dr*/*r*) = *d*(*dr*)/*r *- *dr*/(*r*^2^), i.e. the evolution of the grain strain is established to be given as

(5) $$d\varepsilon =d\left[dr∕r\right]=\left(1+1∕2r\right)D^{2}dt+{\left(a+1\right)}^{2}\left(1+r\right)dN\left(t\right).$$

Notice that in Equation 5, the original grain size, *r*, varies with time. Thus, Equation 5 reflects true strain analysis which is consistent with the fact that grain deformations in nanomaterials may be much larger. Observe that Equations 1 to 5 are given as functions of grain size and time. This implies the *existence of transitional relationships through grain size *between shapes (or form), strain and yield stress. Thus, Equations 1 to 5 are solved simultaneously, using the Engineering Equation Solver software (F-Chart software, Madison, WI53744, USA) for the statistical values of size, shape, strain rate, strain and yield stress after taking the expectations of both sides of each equation. The lognormal probability distribution of grain size in polycrystalline nanomaterials is used, a fact observed both theoretically and experimentally [23-26].

## Results and discussions

It has been acknowledged by another author [13] that there are a number of complications that exist when comparing experimental data from different sources or experiments. This includes, firstly, the fact that the nanomaterials are produced through different processing routes which may lead to different structures, purity and grain size distributions. Secondly, it is remarked [13] that the experimental methods used to impose different strain rates on the specimen often involve widely different loading methods, even in the same study. And thirdly, it is noted [13] that different volumes of materials may be investigated under different experimental techniques. Kumar et al [13] further state that given the small specimen dimensions commonly used to probe many nanocrystalline metals, it is essential to isolate any possible effects of specimen size from intrinsic material properties. The existence of the above complications justifies the variable results reported in the literature. These variable results have equally been revealed in the present work through constraining the mechanisms of grain growth (i.e. constraining processing routes) and the nature of evolution of grain shape. It has been reported that different mechanisms of grain growth can be used (or can be referred to) as different processing routes [9-11]. Because of these varying natures of the results, supported by Kumar et al [13], more emphasis will be put on the discussion of the trends obtained in this paper.

The proposed models are tested with grain growth data from an (nanocrystalline) aluminium sample found in previous publications [[9-11,21-23], and references therein] which are *E *(*r*)_0 _*= *4 nm, *CV *(*r*)_0 _= 0.3, *M*_0_*' *= 0.01 nm^2 ^s^-1^, *v *(*r*, *t*) *= CC/r^m^*, *m = *4, *CC = *12, *a = *0.90, *D = *10^-4^, *< r*_c _*> = *1.95 *< r >*, *h*_0 _= 0.25 nm, *T*_m _(*∞*) = 933.47 K, *H*_m _(*∞*) = 10.71 K J mol^-1^, *σ*_0_*' = *16.7 MPa, *K*_t _= 1.3, *σ*_0 _*= *15.40 MPa, *Kd = *1301.77 MPa nm^1/2^, *R = *8.31 J K^-1 ^mol^-1 ^and *T*_r _*= *300 K. To be consistent with the reports in the literature, the percentage deviations from spherical grains are also dealt with here. The results obtained are presented in the plots below. The results are compared with the results and explanations that other authors have obtained in the literature.

To minimise the cloudy explanation of all the variables presented on the plots, the plots should be interpreted as follows: there are *x*- and *y*-axes used. The plots (e.g. (yield stress) versus (2-D Shape Path_Total_)) indicate variations of the *y*-axis values (e.g. yield stress) as a function of the *x*-axis values (e.g. 2-D Shape Path_Total _(which is when analysis is done following the path of the 2-D shape function due to the TOTAL process)). The variables corresponding to the *y*-axes are labelled on the plots.

**Figure 1:**
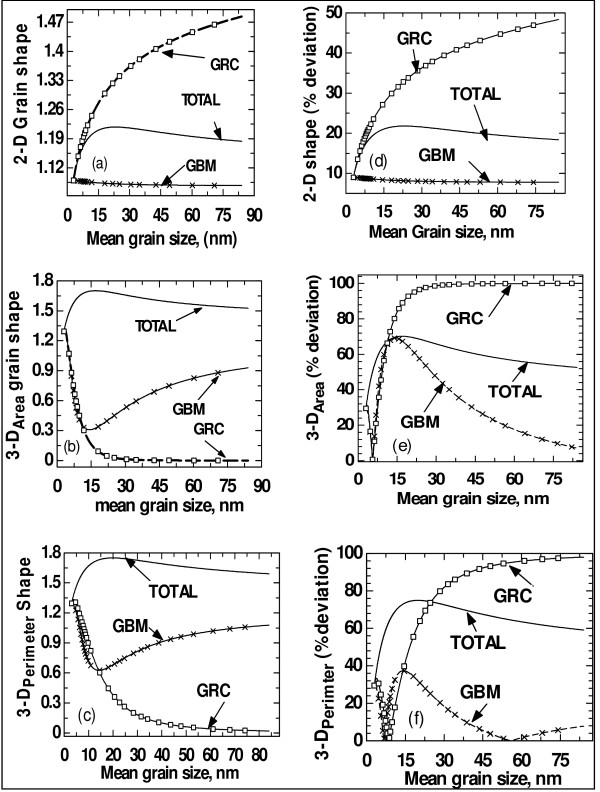
**Evolution as a function of mean grain size**. Evolution as a function of mean grain size of (**a**) 2-D grain shape function, (**b**) 3-D_Area _form function, (**c**) 3-D_Perimeter _form function, (**d**) 2-D percent deviation from spherical shape, (**e**) 3-D_Area _percent deviation from spherical shape and (**f**) 3-D_Perimeter _percent deviation from spherical shape, corresponding to different mechanisms of grain growth (i.e. corresponding to TOTAL process, GBM mechanism and GRC mechanism).

It can be observed from Figure 1 that different mechanisms of grain growth impart different natures of evolution of grain shape. It is remarkably observed that *completely *different natures of evolution of the grain shape are revealed when dealing with 2-D and 3-D analyses (see Figure 1). This is typical of experimental revelation since 2-D analyses are subjects of the orientations of the cutting planes (i.e. cross sections) across the materials on which observations are done, i.e. in 2-D observations, a grain may appear circular from an orientation of the cutting plane, while it might appear to have quite a different shape in another orientation. Although the 3-D form functions used in this report are different (i.e. 3-D_Area _and 3-D_Perimeter_), it is revealed that their 'natures' of evolution are *identical *for the corresponding mechanisms of grain growth (see Figures 1b, c). The differences in the 'extents' to which the different 3-D form functions evolve are revealed in Figures 1e and 1f where their percentage deviations from spherical grains are presented.

It should also be observed that true physical realities (i.e. true since 'shape' depends on *choice *of form function) are revealed in the way that the grain shape evolves with various mechanisms of grain growth or processing routes. The GBM is a grain-curvature-driven process whereby larger grains gradually consume smaller grains through atoms' diffusions at the grain boundaries. This process does not significantly change the shapes of the grains as the grains grow. Thus, the grain shape/form function evolves closer to a spherical shape function of 1 throughout, as revealed in Figure 1. Critical analysis of the percentage deviation from spherical grains in 3-D reveals that there are instances where the grains become spherical before deviating away from the spherical ones when grain growth is due to GBM and GRC only (see Figures 1e, f). This situation of grains becoming spherical at one point and then deviating away from spherical ones observed for GBM and GRC processes (though only slight deviation for GBM only) might be attributed to other grain growth mechanisms (such as T1 event where grains translate and exchange neighbours and T2 events where smaller grains disappear from the system) which are implicitly considered by the GBM and GRC functions [21,22]. The GRC process is one in which grains coalesce, which may (obviously) lead to non-spherical shapes after coalescence. This is reproduced in Figure 1, and it can be seen that the plots of the grain's form due to GRC evolve away from the spherical shape function of 1, showing a maximum of 60% deviation in 2-D grains and 100% deviation in 3-D grains for the given range of grain size less than 90 nm in mean value. Due to the presence of both GBM and GRC mechanisms taking place in most typical nanomaterials, the evolution of the shape function due to the TOTAL process initially deviates away from the spherical shape and finally approaches the spherical shape. The convergence at a later/final stage is due to the fact that a larger amount of energy is required to rotate the grains at larger sizes, thus leading to a diminished GRC process at those sizes [10,21,22].

Comparing the above facts with those from literature, the following are found: Sanders et al. [27] explained that larger grains would deform and work-harden, leading to the rounded shape at the beginning of the stress-strain curve and lower apparent yield strengths compared with the shape curves of GBM only and TOTAL processes. Meyers et al. [14] report that nanocrystalline and ultrafine-grained materials cannot generally sustain uniform tensile elongation and that the increased deviation from spherical grains that is exhibited in some cases comes, basically, from the inhibition of shear localization; the originations of the shape curves in the present report are different from the spherical value of 1. This fact by Meyers et al. [14] simply means that within the region of the RHPR and closer to amorphous materials, the grains are closer to a spherical shape, a fact that has been considered in this report since these shapes' plots start at values that are closer to 1. Meyers et al. [14] explain that GRC processes during grain refinement create larger paths for dislocation movement, causing homogeneous distribution (i.e. equiaxed grain structure) of dislocations which rearrange themselves into elongated dislocation cells; then, as deformation progresses, these cells become elongated subgrains, and finally, these break up into approximately equiaxed micrograins. Meyers et al. [14] further suggest that the relaxation of the broken-down elongated subgrains into an equiaxed microcrystalline structure can occur by minor rotations of the grain boundaries lying along the original elongated boundaries during grain growth.

It should be observed from Figures 1d, e and 1f that nano-sized grains are closer to spherical ones. This observation has also been reported by other authors [13,14,27-32]. Zhang et al. [28] suggested that the convergence to spherical shapes with the reduction of grain size could be an inherent property of nanocrystalline materials given that no porosity and bonding 'were' complete during synthesis. Sanders et al. [27] explain that the calculation of the strain from the uncorrected grip displacement and the possible presence of a few large grains may have contributed to the considerably higher elongations (i.e. deviations from spherical grains) as reported by Nieman et al. [33-35] for nanocrystalline Cu.

**Figure 2:**
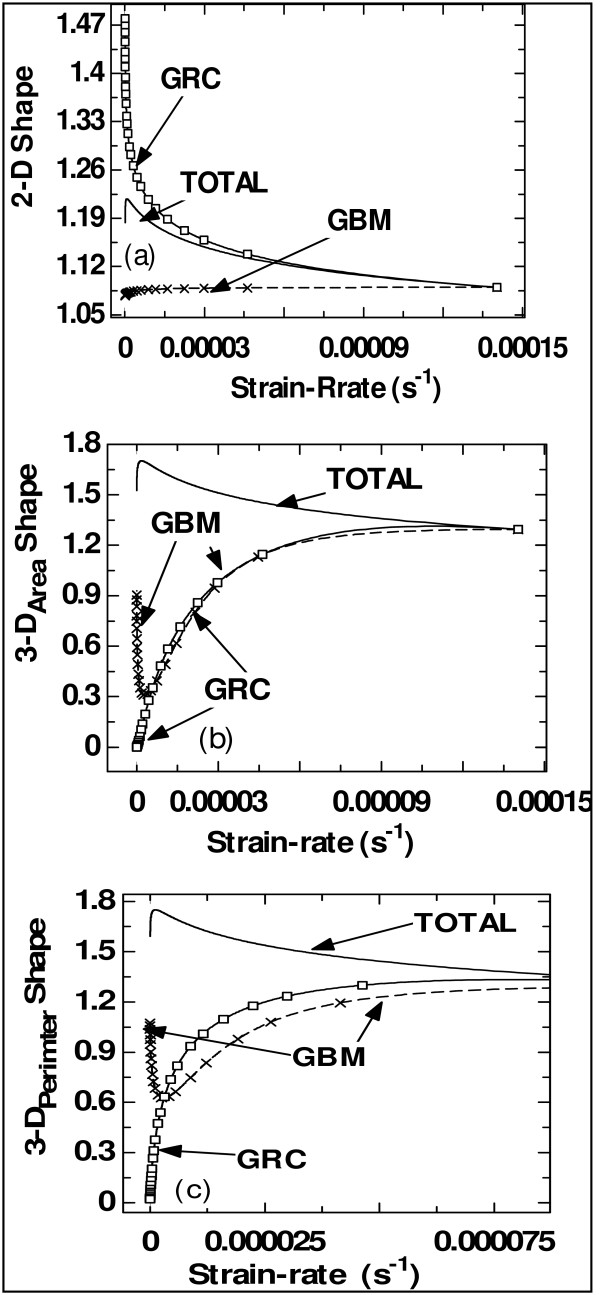
**Evolution as a function of strain rate**. Evolution as a function of strain rate of (**a**) 2-D grain shape function, (**b**) 3-D_Area _form function and (**c**) 3-D_Perimeter _form function, corresponding to different mechanisms of grain growth (i.e. TOTAL process, GBM mechanism and GRC mechanism).

The following factors have been reported to have effects on grain shapes in nanomaterials: work hardening, strain rate sensitivity, thermal softening, contaminates, porosity, processing route and testing method employed to probe rate sensitivity [13,14,29]. It is suggested that [14,29] a way of increasing deviation from spherical grains is by increasing the strain rate with the explanation that this allows the specimen to sustain more plastic strain prior to necking. The effects of strain rate on shape obtained from the present study are presented in Figure 2. These are in agreement with the findings from other authors [13,14], and the results do not agree with the results from Chen et al. [29] since for all the mechanisms of grain growth and all the form functions considered, increase in strain rate leads to the form functions approaching unity (i.e. more grains becoming spherical with increase in strain rate from the present findings). The difference might be due to the fact that grain shape is affected by many factors and only one of them is considered in the models in this report, or it may be due to the processing route followed by Chen et al. [29]. The investigation on the multi-factor effects on grain shape is subject to further research.

Relating grain shape to strain (i.e. studying the effect of shape on strain) does not produce meaningful results. As such, conversely, the effects of strains on shapes are studied. The 'shapes of the plots' obtained are similar to those on the relationship between grain size and shape for various grain growth mechanisms. This simply means that straining has a similar effect on grain shape as that of grain size; this should not be misinterpreted because it is straining that changes grain shapes and grain sizes. This similarity can be explained to be an inherent feature of strain derived from the first principle from grain size. More investigation on other possible definitions of grain strain in nanomaterials and their effects is subject to the author's planned future research. Sanders et al. [27] explain that the difference in elongation-to-failure measurements between their two strain measurement techniques might be due to either machine compliance or non-compliance and acknowledge that, possibly, different definitions may give different results. Another concern in the present paper is that the reported 'strain rate' does not have a direct (or separate) effect on the mechanical properties. Thus, a model of mechanical properties that directly/explicitly includes strain rate parameter may reveal more interesting results.

**Figure 3:**
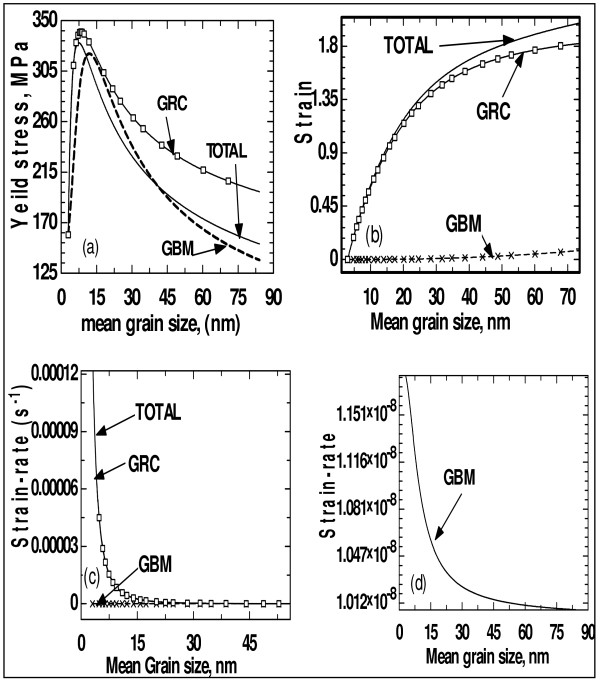
**Evolution as a function of mean grain size**. Evolution as a function of mean grain size of (**a**) yield stress, (**b**) strain, (**c**) strain rate and (**d**) strain rate due to GBM to indicate that the plot varies slowly.

The evolution of mechanical properties as functions of grain size due to the various mechanisms of grain growth is given in Figure 3. It can be observed that different mechanisms of grain growth impart different natures of evolutions on yield stress, strain and strain rate. However, it was observed that the nature (though not the same extent) of evolution of the mechanical properties as functions of mean grain size is the same from 2-D and 3-D observations, probably due to the fact that grain size is valid in all dimensional space. Reporting on the mean grain size in this paper should not be misinterpreted that grain size distribution is not considered. This is because the other parameter of grain size distribution (i.e. grain size dispersion which has been taken into consideration) cannot be explicitly used to 'define' nanomaterials. Since it is observed from Figure 1 that different mechanisms of grain growth impart different natures of evolution of grain shapes, it can be inferred from Figures 1 and 3 that different natures of evolution of the grain shapes impart different natures of evolution on the mechanical properties. Thus, for the sake of projections, the evolution of mechanical properties is 'blindly' modelled using the paths followed by the different shape functions.

In this report, strain rate values are obtained from Equation 5. This is actually the rate of change of strain. The strain rate 'inverse' dependence on grain size revealed in Figures 3c and 3d has been reported in some samples, most especially, when the mean grain size crosses a critical value [13,14,36-38]. It has been explained that there is a critical value that the deformation process changes from one involving GB processes to one dominated by partial dislocations (i.e. a change in the rate controlling mechanism for plastic deformation) [14,36]. Hasnaoui et al. [36] further suggest that the inverse dependence is a geometrical consequence of simulation setup and nanocrystalline structure. It is explained [13,37] that the inverse dependence is because the strain arising from the nucleation and propagation of a dislocation has a limiting length scale equal to that of the grain size. It is reported by Meyers et al. [14] and Gray et al. [39], contrarily, that as grain size increases, the dislocation motion decreases (i.e. high pre-existing dislocation density), boundary diffusion increases and *strain rate increases*. This contradictory report on strain rate is revisited at a later stage in this report.

**Figure 4:**
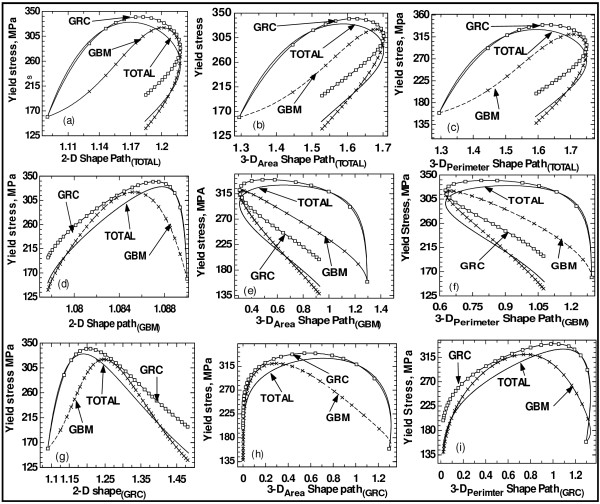
**Evolution of yield stress**. Evolution of yield stress as a function of (**a**) 2-D shape function, (**b**) 3-D_Area _shape function, (**c**) 3-D_Permiter _shape function, (**d**) 2-D_mig _shape function, (**e**) 3-D-_Area_, _mig _shape function, (**f**) 3-D-_Permiter, mig _shape function, (**g**) 2-D_rot _shape function, (**h**) 3-D-_Area, rot _shape function and (**i**) 3-D-_Permiter, rot _shape function. Where, for example, 3-D-_Permiter, mig _implies that obtained results are for a system that is analysed following the path of the 3-D shape function due to GBM processes only.

Results on the relationships between *percentage *deviation from spherical grains in 3-D and yield stress due to GRC and GBM processes are not informative because of the fact that the curves of their form functions cross the spherical value of 1. Thus, these plots on the relationship between percentage deviations and mechanical properties are not presented here. It should be observed from Figure 4 that different mechanisms of grain growth (i.e. different processing routes) as well as different grain shape functions impart different natures of evolution on yield stress. It should be observed that if grains were to evolve following the shapes determined by the path of the TOTAL process, then the plots for this relationship between shape and yield stress will be similar for all the mechanisms of grain growth. These plots do not reveal simple and meaningful functions though a similar shape of the plots has been obtained by Kumar et al. [13]. If grains were to evolve following the shapes determined by the path of the 3-D shape function due to GBM only, then plots of the shape-to-property relationship will not also be simple-to-explain functions though the trend is different from that of the path followed by the TOTAL process. It has been equally reported in the literature [13,14] that contradictory reports exist about the dependences of nanomaterials' mechanical properties on the internal structures.

The trend revealed by Figures 4d, g, h and 4i is similar to that given by the RHPR-to-HPR trend whereby as grain growth proceeds and the grains deviated from spherical grains, the yield stress increases reaching maximum value at a critical deviation (though not unique) where the yield stress starts decreasing with further deviation from the spherical shape (i.e. with further elongation). It should be noted that both trends of increasing and decreasing mechanical properties with increasing deviation from spherical grains have been observed by Meyers et al. [14]. Meyers et al. [14] and Sanders et al. [27] also suggested that both properties and elongation (deviation from spherical grains) depend on the processing route, a fact that has been revealed in the results obtained under the proposed approach. Most authors have reported that nanocrystalline materials have higher yield stresses, and their grains are closer to a spherical shape [13,30-33]. Dalla et al. [30] showed that the shapes of the tensile stress-strain curve and tensile elongation depend on specimen size, with uniform deformation along the gauge section observed in small specimens, but not in larger specimens. The observed differences were attributed to microstructural inhomogeneity [13,30].

**Figure 5:**
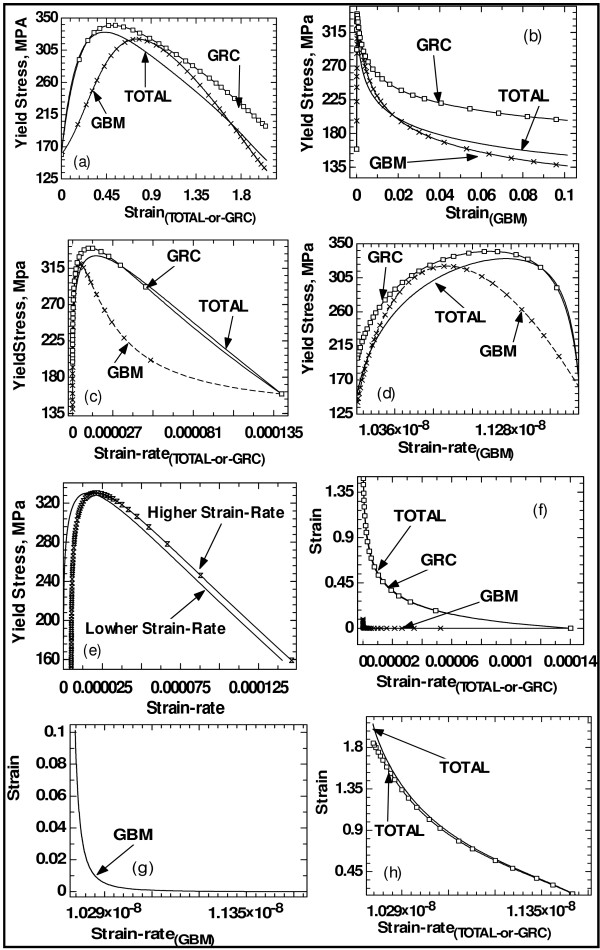
**Evolution of yield stress as a function of strain/strain rate**. Evolution of (**a**) yield stress as a function of strain due to TOTAL and GRC processes, (**b**) yield stress as a function of strain due to GBM process, (**c**) yield stress as a function of strain rate due to TOTAL and GRC processes, (**d**) yield stress as a function of strain rate due to GBM process, (**e**) yield stress as a function of varying strain rate, (**f**) strain as a function of strain rate for TOTAL, GBM and GRC processes, (**g**) strain as a function of strain rate as a result of GBM processes to indicate that it decays slowly with increasing strain rate and (**h**) strain as a function of strain rate to indicate the values as a result of TOTAL and GRC processes are not the same.

Results on the relationship between stress and strain, stress and strain rate, and strain and strain rate are shown in the Figure 5. These results also depend on the mechanisms of grain growth and the way that the form function evolves. The general trend is similar to that of the RHPR-to-HPR trend. Thus, there exist critical strain and critical strain rate beyond which further increases do not lead to increase in yield stress of the materials. Similar results have been obtained and reported in the literature [13,14]. In fact, Kumar et al. [13] observed that material damage/failure initiates when the local plastic strain reaches a critical value, and the material strength declines linearly with plastic strain to zero over a certain plastic strain increment. It is reported that the stress-strain response of a nanocrystalline metal under tension shows a rapid peak and subsequent softening [14,40] due largely to necking with the rapid peak, indicating localised deformation [14]. It is reported that experimental studies have shown that nanocrystalline metals exhibit higher mechanical response with higher strain-rate [13,30,41,42].

Kumar et al. [13] and Meyers et al. [14] remarked that no clear trends could be established on the effects of strain rate on tensile strain to failure as the mechanisms underlying the unusual rate sensitivity of deformation of nanocrystalline metals and alloys are not fully understood at this time, i.e. there are conflicting results in the literature on strain rate sensitivity. It has been observed [13,43,44] that the strain-to-failure mechanism increases markedly with increasing strain rate, and it is reported [13] that this trend is distinctly different from that of conventional microcrystalline copper where the fracture strain is known to drop slightly with strain rate. Contrary to that, Meyers et al. [14] state that there have been reports of both increased and decreased strain rate sensitivity with decreasing grain size in metals. Both increasing and decreasing trends have been revealed in Figure 5 from the present work.

It can be concluded, in line with Meyers et al. [14], that grain size, grain shape, strain rate sensitivity and deformation mechanisms are connected, and it is possible through the manipulation of the nanostructure to increase deviation from spherical shapes and mechanical properties. Furthermore, it can be said that although all these mechanisms of plastic deformation may play a role under specific internal (grain size, composition, etc.) or external (temperature, strain rate, stress state) conditions, a few of them seem dominant in the nanocrystalline regime [18]. An observation that could not be explained in this report is whether it could be inferred that as the form functions evolve above or below the value of 1, it implies that the grains are getting convex or concave or vice versa.

## Conclusion

An approach on simultaneously considering grain shape during modelling and designing of nanomaterials' properties has been proposed and tested. The way forward has been to start with the study of the nature of evolution of the shape function or form. It is found in this paper that the long-run results reveal that GBM process causes grains to become spherical during grain growth, GRC makes them deviate away from becoming spherical, and they initially deviate away from becoming spherical before converging into spherical ones due to the TOTAL process. Percentage deviations from spherical grains depend on dimensional space and form: a 0% minimum and a 100% maximum deviations were observed. It is shown that the plots for grain shape functions lie above the spherical (control) value of 1 in 2-D grains for all considered grain growth mechanisms. Some plots lie above the spherical value, and others approach the spherical value before deviating below it when dealing with 3-D grains. The physical interpretations of these variations are explained from elementary principles about the different grain growth mechanisms. It can be concluded that the nature of evolution of the form depends on the choice of the form function, dimensional space and processing routes (i.e. grain growth mechanisms). An observation that could not be explained in this report is whether the evolution of the form function above or below the value of 1 implies that the grains are getting convex or concave or vice versa.

The variable results reported in the literature about the natures of evolution of mechanical properties have equally been revealed in the present work through constraining the mechanisms of grain growth and the nature of evolution of grain shape and by considering all the statistics of the grain size distribution. It can be concluded that mechanical properties of nanomaterials depend simultaneously on the internal structure morphology (shape), the grain size distribution and processing routes. It has been observed that there exist some critical points beyond which the interrelationships between nanomaterials' mechanical properties change drastically; for example, there exist critical mean grain size, critical strain, critical strain rate, critical grain shape, etc. beyond which the HRP changes to RHPR. It is also observed in the present findings that materials whose grains deviate further away from spherical ones have more enhanced properties, while materials with spherical grains have lesser properties. It can be concluded that constraining the nature of evolution of the form function leads to the design of nanomaterials with different mechanical properties.

## Abbreviations

GB: grain boundary; GBM: curvature driven grain boundary migration; GRC: mis-orientation angle driven grain rotation coalescence; HPR: Hall-Petch Relationship; RHPR: Reverse Hall-Petch Relationship; TOTAL: simultaneous occurrence of GRC and GBM mechanisms.

## Competing interests

The authors declare that he has no competing interests.

## Authors' contributions

This paper has been prepared by a sole author; TBT. TBT conceptualized and designed the study, established and ran the model equations, obtained the data, compared results with those from the literature and prepared the manuscript
